# Supplementary material for: Direct Disk Diffusion Testing and Antimicrobial Stewardship for Gram-Negative Bacteremia in the Context of High Multidrug Resistance
Source: Antibiotics (Basel). 2025 Jul 19;14(7):726. doi: 10.3390/antibiotics14070726 (PMC12291970; doi:10.3390/antibiotics14070726)
Supplement: Supplementary file 1 [file antibiotics-14-00726-s001.zip › antibiotics-3734021-supplementary.pdf]

Table S1. Empirical and definitive antibiotic therapies

**Empirical antibiotic**<br>N=188 (%)	**Definitive antibiotic**<br>N=179 (%)
Meropenem 68 (36.2)	Ceftriaxone 44 (24.6)
Piperacillin/tazobactam 29 (15.4)	Meropenem 43 (24.0)
Ceftriaxone 21 (11.2)	Colistin 27 (15.1)
Colistin 16 (8.5)	Cefoperazone/sulbactam 9 (5.0)
Ceftazidime 15 (8.0)	Ceftazidime 7 (3.9)
Levofloxacin 7 (3.7)	Fosfomycin 7 (3.9)
Metronidazole 7 (3.7)	Levofloxacin 6 (3.4)
Others[a] 25 (13.3)	Ertapenem 6 (3.4)
	Others[b] 30 (16.8)

[a]Others are cefoperazone/sulbactam, co-amoxiclav, amikacin, imipenem, fosfomycin, ceftazidime/avibactam, vancomycin, doxycycline, sitafloxacin, cefazolin, gentamicin, azithromycin

[b]Others are ciprofloxacin, piperacillin/tazobactam, imipenem, tigecycline, ceftazidime/avibactam, vancomycin, cefoperazone/sulbactam, metronidazole, sitafloxacin, ampicillin/sulbactam, clindamycin

Table S2. Accuracy of direct disk diffusion compared to standard antibiotic susceptibility testing

No.	Organism	Susceptibility result																				
		Cefotaxime			Cefoperazone/sulbactam			Ceftazidime/avibactam			Ertapenem			Imipenem			Sitafloxacin			Fosfomycin		
		DD (mm)	Standard AST	Concordance*	DD (mm)	Standard AST	Concordance	DD (mm)	Standard AST	Concordance	DD (mm)	Standard AST	Concordance	DD (mm)	Standard AST	Concordance**	DD (mm)	Standard AST	Concordance	DD (mm)	Standard AST	Concordance
1	*Escherichia coli*	8	R	CA	26	S		28	S		28	S		28	S	CA	20	S		28	S	
2	*Pseudomonas aeruginosa*	ND	NA	NA	21	S		28	S		6	NA		25	S	CA	25	S		25	NA	
3	*Escherichia coli*	15	R	CA	28	S		28	S		30	S		28	S	CA	28	S		28	S	
4	*Klebsiella pneumoniae*	ND	R	NA	ND	NA		ND	NA		ND	S		ND	S	NA	ND	NA		ND	NA	
5	*Escherichia coli*	6	R	CA	6	R		19	R		6	R		16	I	mE	11	R		28	S	
6	*Klebsiella pneumoniae*	6	R	CA	15	R		25	S		13	R		31	S	CA	9	R		6	R	
7	*Escherichia coli*	13	R	CA	27	S		27	S		32	S		28	S	CA	23	S		28	S	
8	*Klebsiella pneumoniae*	6	R	CA	18	I		24	S		8	R		20	R	mE	15	I		18	S	
9	*Klebsiella pneumoniae*	6	R	CA	8	R		28	S		17	R		6	I	mE	6	R		6	R	
10	*Acinetobacter baumannii*	6	R	CA	22	S		13	NA		ND	NA		11	R	CA	22	S		12	NA	
11	*Escherichia coli*	13	R	CA	28	S		30	S		32	S		28	S	CA	20	S		28	S	
12	*Klebsiella pneumoniae*	9	S	ME	30	S		30	S		32	S		30	S	CA	30	S		22	S	
13	*Klebsiella pneumoniae*	6	R	CA	8	R		21	S		6	R		18	R	CA	8	R		6	R	
14	*Klebsiella pneumoniae*	27	S	CA	28	S		28	S		30	S		28	S	CA	29	S		18	S	
15	*Acinetobacter baumannii*	20	S	CA	28	S		22	NA		ND	NA		32	S	CA	35	S		6	NA	
16	*Enterobacter cloacae*	6	R	CA	8	R		14	R		9	R		15	R	CA	17	S		14	I	
17	*Acinetobacter baumannii*	6	R	CA	12	R		6	NA		ND	NA		6	R	CA	20	S		13	NA	
18	*Klebsiella pneumoniae*	6	R	CA	12	R		22	S		9	R		19	R	CA	15	I		16	S	
19	*Pseudomonas aeruginosa*	10	NA	NA	19	I		24	S		6	NA		14	R	CA	25	S		6	NA	
20	*Salmonella* serogroup B	28	S	CA	26	NA		29	NA		33	S		27	S	CA	38	NA		25	NA	
21	*Acinetobacter baumannii*	6	R	CA	6	R		6	NA		ND	NA		6	R	CA	22	S		10	NA	
22	*Escherichia coli*	11	R	CA	26	S		28	S		29	S		26	S	CA	23	S		26	S	
23	*Klebsiella pneumoniae*	6	R	CA	6	R		18	R		8	R		17	R	CA	19	S		28	S	

24	*Escherichia coli*	14	R	CA	28	S	[redacted]	29	S	[redacted]	30	S	[redacted]	30	S	CA	18	S	[redacted]	28	S	[redacted]
25	*Escherichia coli*	24	S	CA	29	S	[redacted]	32	S	[redacted]	36	S	[redacted]	30	S	CA	29	S	[redacted]	30	S	[redacted]
26	*Klebsiella pneumoniae*	13	R	CA	26	S	[redacted]	28	S	[redacted]	30	S	[redacted]	32	S	CA	26	S	[redacted]	22	S	[redacted]
27	*Klebsiella pneumoniae*	27	S	CA	30	S	[redacted]	27	S	[redacted]	29	S	[redacted]	24	S	CA	26	S	[redacted]	20	S	[redacted]
28	*Escherichia coli*	20	R	mE	23	S	[redacted]	27	S	[redacted]	28	S	[redacted]	26	S	CA	18	S	[redacted]	24	S	[redacted]
29	*Acinetobacter baumannii*	6	R	CA	21	S	[redacted]	13	NA	[redacted]	ND	NA	[redacted]	11	R	CA	23	S	[redacted]	12	NA	[redacted]
30	*Escherichia coli*	30	S	CA	28	S	[redacted]	29	S	[redacted]	28	S	[redacted]	28	S	CA	30	S	[redacted]	24	S	[redacted]
31	*Escherichia coli*	27	S	CA	26	S	[redacted]	28	S	[redacted]	29	S	[redacted]	23	S	CA	24	S	[redacted]	24	S	[redacted]
32	*Escherichia coli*	6	R	CA	21	S	[redacted]	27	S	[redacted]	25	S	[redacted]	23	S	CA	18	S	[redacted]	24	S	[redacted]
33	*Escherichia coli*	32	S	CA	32	S	[redacted]	33	S	[redacted]	35	S	[redacted]	33	S	CA	25	S	[redacted]	30	S	[redacted]
34	*Escherichia coli*	30	S	CA	25	S	[redacted]	25	S	[redacted]	28	S	[redacted]	24	S	CA	24	S	[redacted]	25	S	[redacted]
35	*Klebsiella pneumoniae*	19	R	CA	25	S	[redacted]	26	S	[redacted]	28	S	[redacted]	31	S	CA	10	R	[redacted]	21	S	[redacted]
36	*Proteus mirabilis*	30	S	CA	28	S	[redacted]	30	S	[redacted]	31	S	[redacted]	28	S	CA	26	S	[redacted]	30	S	[redacted]
37	*Escherichia coli*	28	S	CA	25	S	[redacted]	28	S	[redacted]	29	S	[redacted]	28	S	CA	27	S	[redacted]	25	S	[redacted]
38	*Escherichia coli*	6	R	CA	18	I	[redacted]	28	S	[redacted]	24	S	[redacted]	26	S	CA	23	S	[redacted]	25	S	[redacted]
39	*Escherichia coli*	27	S	CA	24	S	[redacted]	27	S	[redacted]	28	S	[redacted]	25	S	CA	30	S	[redacted]	22	S	[redacted]
40	*Klebsiella pneumoniae*	25	S	CA	25	S	[redacted]	28	S	[redacted]	26	S	[redacted]	24	S	CA	30	S	[redacted]	14	I	[redacted]
41	*Burkholderia pseudomallei*	ND	R	NA	29	NA	[redacted]	ND	NA	[redacted]	ND	R	[redacted]	ND	S	NA	ND	NA	[redacted]	ND	NA	[redacted]
42	*Acinetobacter baumannii*	18	S	mE	28	S	[redacted]	22	NA	[redacted]	18	NA	[redacted]	26	S	CA	27	S	[redacted]	9	NA	[redacted]
43	*Escherichia coli*	9	S	ME	30	NA	[redacted]	32	NA	[redacted]	36	S	[redacted]	30	S	CA	36	NA	[redacted]	28	NA	[redacted]
44	*Klebsiella pneumoniae*	30	S	CA	30	S	[redacted]	28	S	[redacted]	32	S	[redacted]	29	S	CA	29	S	[redacted]	22	S	[redacted]
45	*Pseudomonas aeruginosa*	11	NA	NA	ND	NA	[redacted]	28	S	[redacted]	ND	NA	[redacted]	27	S	CA	33	S	[redacted]	13	NA	[redacted]
46	*Klebsiella oxytoca*	ND	S	NA	ND	NA	[redacted]	31	S	[redacted]	31	S	[redacted]	29	S	CA	30	S	[redacted]	18	S	[redacted]
47	*Escherichia coli*	10	R	CA	ND	NA	[redacted]	26	NA	[redacted]	28	S	[redacted]	27	S	CA	25	NA	[redacted]	26	NA	[redacted]
48	*Acinetobacter baumannii*	6	R	CA	ND	NA	[redacted]	ND	NA	[redacted]	ND	NA	[redacted]	18	R	VME	21	S	[redacted]	11	NA	[redacted]
49	*Klebsiella pneumoniae*	31	S	CA	ND	NA	[redacted]	26	S	[redacted]	31	S	[redacted]	28	S	CA	28	S	[redacted]	16	S	[redacted]
50	*Escherichia coli*	12	R	CA	ND	NA	[redacted]	28	S	[redacted]	31	S	[redacted]	29	S	CA	20	S	[redacted]	32	S	[redacted]

51	*Acinetobacter baumannii*	6	R	CA	ND	NA		6	NA		ND	NA		6	R	CA	22	S		10	NA	
52	*Klebsiella pneumoniae*	6	R	CA	ND	NA		23	S		26	S		27	S	CA	14	I		18	S	
53	*Escherichia coli*	6	R	CA	ND	NA		30	S		28	S		30	S	CA	24	S		28	S	
54	*Klebsiella pneumoniae*	30	S	CA	ND	NA		30	S		31	S		28	S	CA	30	S		18	S	
55	*Salmonella* serogroup D	31	S	CA	ND	NA		30	NA		32	S		32	S	CA	31	NA		25	NA	
56	*Pseudomonas aeruginosa*	20	NA	NA	ND	NA		28	S		25	NA		30	S	CA	32	S		13	NA	
57	*Escherichia coli*	25	S	CA	ND	NA		28	S		29	S		25	S	CA	23	S		25	S	
58	*Escherichia coli*	28	S	CA	ND	NA		28	S		30	S		28	S	CA	ND	NA		24	S	
59	*Acinetobacter baumannii*	6	R	CA	ND	NA		15	NA		ND	NA		12	R	CA	ND	NA		11	NA	
60	*Escherichia coli*	27	S	CA	ND	NA		28	S		33	S		28	S	CA	ND	NA		27	S	
61	*Escherichia coli*	28	S	CA	ND	NA		28	S		32	S		28	S	CA	ND	NA		27	S	
62	*Escherichia coli*	27	S	CA	ND	NA		28	S		30	S		23	S	CA	21	S		26	S	
63	*Escherichia coli*	10	R	CA	ND	NA		30	S		30	S		30	S	CA	28	S		30	S	
64	*Aeromonas hydrophila/caviae*	30	S	CA	ND	NA		27	NA		23	S		22	I	CA	30	NA		ND	NA	
65	*Acinetobacter baumannii*	6	R	CA	ND	NA		6	NA		ND	NA		6	R	CA	20	S		ND	NA	
66	*Escherichia coli*	30	S	CA	ND	NA		30	S		30	S		30	S	CA	30	S		ND	S	
67	*Escherichia coli*	28	S	CA	ND	NA		30	S		34	S		30	S	CA	21	S		ND	NA	
68	*Escherichia coli*	35	S	CA	ND	NA		32	NA		35	S		30	S	CA	28	NA		ND	S	
69	*Escherichia coli*	6	R	CA	ND	NA		10	R		6	R		12	R	CA	11	R		ND	S	
70	*Klebsiella pneumoniae*	40	S	CA	ND	NA		38	S		40	S		38	S	CA	38	S		ND	S	
71	*Klebsiella pneumoniae*	6	R	CA	ND	NA		23	S		22	S		25	S	CA	16	S		ND	S	
72	*Klebsiella pneumoniae*	34	S	CA	ND	NA		28	S		32	S		28	S	CA	34	S		ND	S	
73	*Pseudomonas aeruginosa*	20	NA	NA	ND	NA		30	S		ND	NA		25	S	CA	30	S		ND	NA	
74	*Burkholderia cepacia* complex	20	NA	NA	ND	NA		28	NA		15	NA		16	NA	NA	28	NA		ND	NA	

Abbreviation: CA, categorical agreement; I, intermediate; mE, minor error; ME, major error; NA, not applicable; ND, not done; R, resistance;S, susceptible; VME, very major error

CA was defined by identical results between direct disk diffusion (DD) test and standard culture.
ME referred to false resistance (resistant by DDT and susceptible by standard culture), whilst VME (very major error) indicated false susceptibility (susceptible by DDT and resistant by standard culture).
All other mismatches between DDT and standard culture that differed by one susceptibility category were classified as mE.
*, **Retrospective interpretation using the 2025 CLSI diameter breakpoints for ceftriaxone and meropenem, respectively.
